# **Habitability at the edge of the redox boundary during the Permian**–**Triassic mass extinction**

**DOI:** 10.1038/s41598-026-47893-w

**Published:** 2026-04-15

**Authors:** Borhan Bagherpour, Omid H. Ardakani, Daniel Herwartz, Stephen E. Grasby

**Affiliations:** 1 Institute of Geosciences, Ruhr University Bochum, Universitätsstraβe 150, 44801 Bochum, Germany; 2 Natural Resources Canada, Geological Survey of Canada, 3303 33rd St. NW, Calgary, AB T2L 2A7 Canada

**Keywords:** Permian, Triassic, Mass extinction, Redox condition, Tethys, Biogeochemistry, Climate sciences, Ecology, Ecology, Environmental sciences, Microbiology, Ocean sciences, Solid Earth sciences

## Abstract

Global superanoxia is widely accepted as one of the main drivers of the end–Permian Mass Extinction (EPME) alongside, oceanic acidification, productivity collapse, and toxification. However, modeling and paleontological studies suggest spatial heterogeneity, with parts of the Tethys Ocean remaining oxygenated. To assess water–column oxygenation in the central Tethys, we studied two shallow–marine Permian–Triassic sections in equatorial paleolatitudes of central Iran; one with terrestrial input, the other fully marine. Continuous sedimentation across the EPME enables reconstruction of the latest Permian environment. U, Th, Mo, and Mn concentration data indicate well–oxygenated conditions until the EPME horizon, followed by Mn concentration peaks in microbialite/black shale intervals that reflect fluctuating oxic–anoxic conditions across the EPME. Micronutrient decline preceding the extinction suggests reduced local productivity. Thus, oxic conditions in microbialite–bearing shallow–marine settings were likely sustained by photosynthetic O₂ production and/or wave agitation. Low productivity also implies limited oxygen demand for organic matter remineralization, minimizing redox stress in these environments. We highlight shallow–marine Tethyan settings as potential oxygenated habitat during deep–sea anoxia, although a fluctuating chemocline repeatedly introduced Mn into marine environments, restricting oxidized habitat to the surface layer in contact with the atmosphere and/or oxygen–producing microbial mats.

**Supplementary Information:**

The online version contains supplementary material available at 10.1038/s41598-026-47893-w.

## Introduction

During the end–Permian mass extinction (EPME) ~ 90% of marine species become extinct^1^. This extinction has been dated between 251.941 ± 0.037 and 251.880 ± 0.031 Ma^2^ and (251.94 ± 0.03 Ma in Dongpan and Penglaitan, S. China)^3,4^. The EPME is associated with rapid equatorial warming from 21 to 36 °C as recorded in China^5^ and 25 to 35 °C in the Abadeh section (Central Iran), over a maximum duration of ~ 37 kyr^6^. A 12 °C gradual warming is also documented in the Aras Vally section (Northwest Iran)^7^. This rapid climate warming is considered to be caused by greenhouse gas emissions from the Siberian Traps^5^, which induced water stratification and a “global superanoxia event” commonly attributed as one of the main kill mechanisms of the EPME^8,9^. In addition to anoxia, oceanic acidification [e.g.^10,11^], productivity collapse [e.g.^12,13^], as well as metal and coal fly ash toxification [e.g.^14–16^] are suggested to have impacted marine ecosystems.

The prevalence of anoxia is well documented in deep–marine settings of northern Pangea^17^, the Tethyan realm deep–slope^18,19^, and the Panthalassa Ocean^20,21^. However, oxygenated conditions based on geochemical studies are inferred for shallow–marine settings in the central Tethys Ocean^22,23^, as well as NW Pangea^24^. Uranium concentration and isotopic composition studies suggest that anoxic conditions only expanded from 0.2 to 20%^25^ or 17 to 60%^26^ of the seafloor. A considerable portion of the seafloor thus remained oxygenated across the extinction horizon, although the respective constraints on timescales do not resolve potential rapid changes in oxygenation occurring during the main EPME. In addition to site–specific studies, global models of Permian–Triassic Boundary (PTB) seafloor O_2_ levels indicate severe anoxia in the Panthalassa Ocean (intensified in high latitudes), but an elevated O_2_ concentration in tropical regions, specifically in the Tethyan realm^27^. This model suggests the depletion of surface nutrients at high latitudes, leading to a decrease in nutrient supply to the tropical oceans (i.e., Tethys), and as observed in NW Pangea^13,28^. The modeled reduction in nutrients in the Tethys decreases the rate of O_2_ consumption associated with oxidation of organic matter (OM), which is suggested to limit the spread of anoxia^27^. These observations suggest that the end–Permian anoxia was a heterogeneous phenomenon and that it was not global. Thus, global redox variability remains under-constrained. Here we test the extent of anoxia by investigating sedimentary sections from the part of the central Tethys that was modelled to be the most oxygenated part of any oceanic realm during the EPME^27^.

**Fig. 1:**
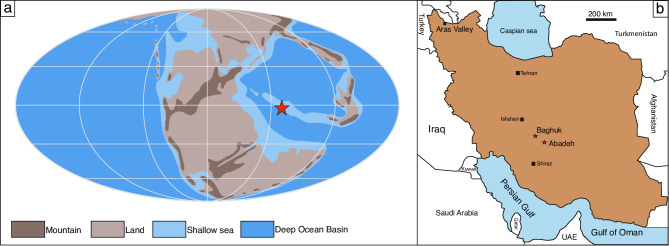
Maps of the studied sections. **a**, Paleogeographic map of the Early Triassic modified from (http://www.scotese.com)^33^. **b**, Geographic map of Iran. Modified from Natural Earth (public domain; https://www.naturalearthdata.com/). Stars indicate the location of the studied sections. Figures created using CorelDRAW, Version 26.2.0.170.

In shallow–marine environments of the Tethys the PTB is commonly characterized by microbial limestones, the base of which is associated with a substantial unconformity. In deeper–marine sections such as Aras Valley (Iran) and Shangsi (China) (where microbialite build–ups are absent) no unconformity is documented^3,29^. Unlike other microbialite–bearing sections, field observations and biostratigraphic studies represent the classical Abadeh section (central Iran, Fig. 1a, b) as a well–preserved PTB section with no, or only minor, hiatus^6,30^. The continuous sedimentation, despite eustatic sea–level drop, was caused by tectonic subsidence related to rifting during the Permian–Triassic interval^31,32^. Such a well–preserved archive in the central Tethys Ocean offers an excellent opportunity to reconstruct the paleoredox conditions in the equatorial eastern Tethys during the EPME. Furthermore, the nearby Baghuk section with black shale deposited across the PTB provides a comparison of local redox conditions in a contrasting depositional setting, with significant terrestrial input.

In this study, we conduct a detailed field investigation and measure the concentration of major, trace, and rare earth elements plus yttrium (REE + Y), along with carbonate carbon isotope (δ^13^C_carb_) and strontium isotope ratio, and total organic carbon (TOC) content from the Abadeh (Table S1) and Baghuk (Table S2) sections. These data allow evaluation of the oxygenation state of the shallow–marine environments. Investigating the same stratigraphic interval in two sections, with contrasting sedimentary regimes, supports a robust reconstruction of regional oxygenation state in the central Tethys. We document strong nutrient drawdown during the EPME that lowered O_2_ demand in lower latitudes, as proposed by the model of Penn et al.^27^. We suggest that locally produced O_2_ by cyanobacterial growth, and/or air O_2_ dissolution due to wave action, resulted in a well–oxygenated shallow water environment. However, brief episodes of anoxic conditions are also evident suggesting that this last refugium for O_2_ metabolizing marine organisms was still exposed to heavy stress.

## Studied sections and stratigraphy

**Fig. 2:**
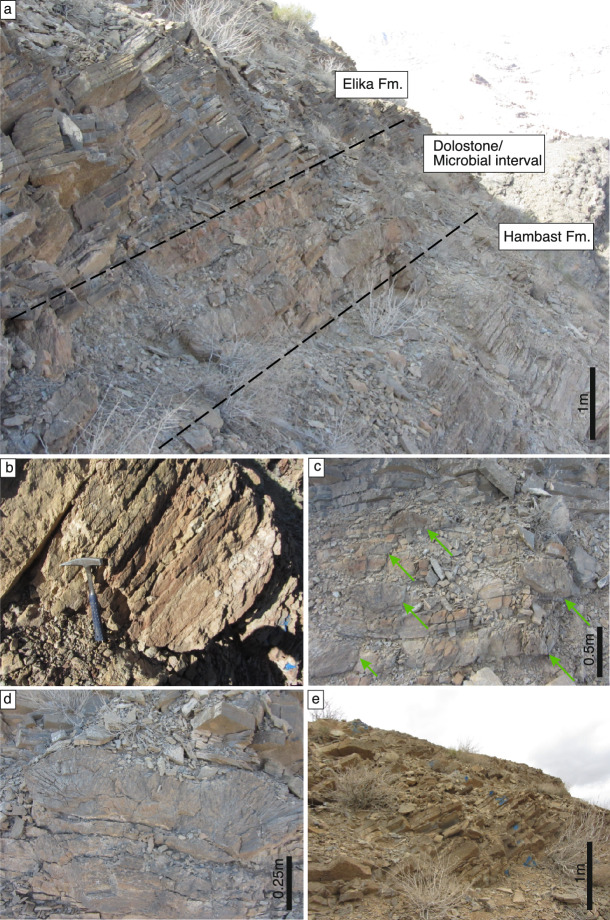
Field photographs of the Abadeh section. **a**, Outcrop photograph of the Permian Hambast and Triassic Elika formations. **b**, Close–up picture of the nodular limestone in the upper part of the Hambast Fm. **c**, Intercalation of dolostone beds and microbialite build ups (green arrows). For an enlarged view see Supplemental Figure S1. **d**, Digitated microbialite build up. For an enlarged view see Supplemental Figure S1. **e**, Thin–bedded dark grey limestone in the base of the Elika Fm.

Both the Abadeh (30°53′43"N; 53°12′15"E) and Baghuk (31°34′00"N; 52°26′37"E) sections were located on the northern Neo–Tethyan shelf (Cimmerian Microcontinent) near the equator during the PTB (Fig. 1a, b)^34^. The Late Permian Hambast Formation (Fm.) in the Abadeh section is mainly composed of ammonoid–rich, nodular, medium–bedded limestone (Fig. 2a, b). The upper most 3.8 m of the Hambast Fm. contains abundant *Paratirolites* ammonite and accordingly it is commonly referred to as “Paratirolites limestone” in the literature (e.g.^6^). The Hambast Fm. is conformably replaced by a 140 cm–thick microbial build–up intercalated with thin–bedded dolostone layers (Fig. 2c, d). This microbial limestone/dolostone interval is, in turn, sharply overlain by thin–bedded dark grey limestones of the Elika Fm. (Fig. 2e).

**Fig. 3:**
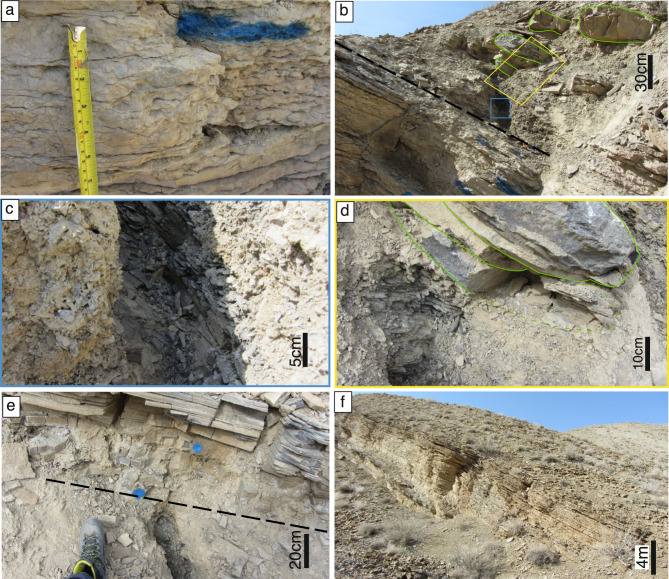
Field photographs of the Baghuk section. **a**, Close–up picture of the nodular limestone (Paratirolites limestone) in the uppermost part of the Hambast Fm. **b**, Exposure of the Hambast Fm. and conformably overlying intercalating black shale and microbialite build ups (Baghuk Member). Green lines outline the microbialite build ups. **c**, Close up picture of black shales in the blue rectangle in Fig. 3b. **d**, Close up picture of black shales in the yellow rectangle in Fig. 3b. Green lines outline the microbialite build ups. **e**, Boundary between black shale (of Baghuk Member) and thin–bedded dark grey limestone in the base of the Elika Fm. **f**, Landscape view of thin–bedded limestone in the base of Elika Fm.

The Permian Hambast Fm. in the Baghuk section (Fig. 3a, b) is lithologically identical to the Abadeh section but in the uppermost part it is capped by a 2–m–thick interval of dark grey shale, intercalated with microbialite (Baghuk Member) (Fig. 3c, d). The Triassic Elika Fm. exhibits similar lithological characteristics (Fig. 3e, f) to those observed in Abadeh. The topmost surface of the Hambast Fm. does not exhibit any erosional features in either section.

**Fig. 4:**
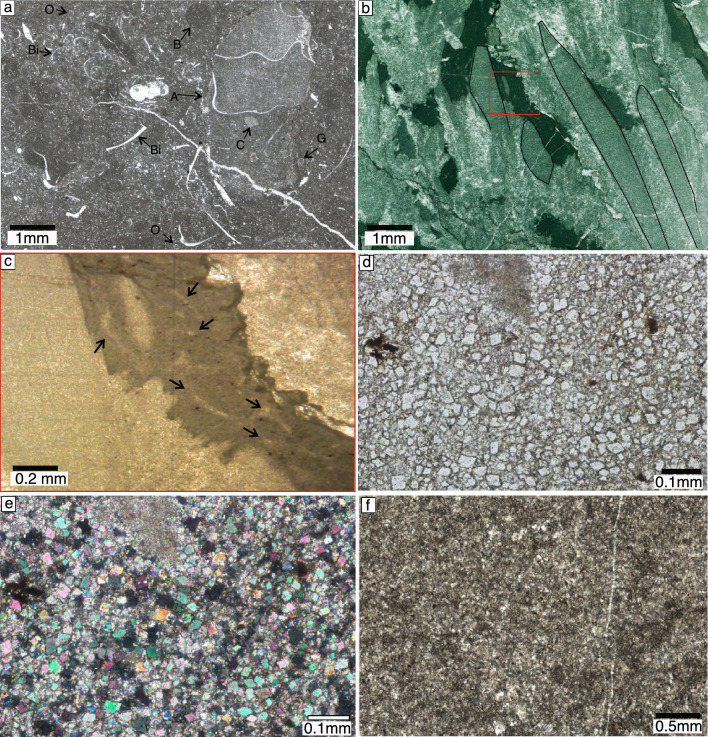
Photomicrographs of microfacies of Hambast and Elika formations. **a**, Bioclastic limestone of the Paratirolites limestone (Baghuk section). A; ammonoid, B; bioturbation, C; Crinoid, Bi; Bivalve, G; Gastropod, O; Ostracod, Br; Brachiopod. **b**, Finely laminated stromatolite (needle-like) columns of digitated microbialites overgrown by calcite fans of the Elika Fm. (Abadeh section). **c**, Enlargement of red rectangle in Fig. 4b. black arrows indicate sponge fibers in the in the sediment between the columns. **d**, dolostone intercalating with microbialite built ups (Natural light, Abadeh section). **e**, Cross–polarized light micrograph of Fig. 4d. **f**, Recrystallized limestone of thin–bedded limestone of the Elika Fm. overlying the microbialite unit (Abadeh section).

At microscopic scale, the Paratirolites limestone (in both sections) represent a mud supported wackestone texture containing abundant sponge spicules. Ammonoids, thin–shelled bivalves, ostracods, crinoids, radiolarian and rare foraminifera constitute the bioclasts of this interval (Fig. 4a). Bioturbation is occasionally present.

Microbial build ups represent digitated and clotted structures. Elongated branching thin stromatolite columns which are both straight and bended into macro and microscopic scales are characterized by laminations (Fig. 4b). Abiotic upward branching overgrowth cements are abundant on the periphery and top of the stromatolite needle–like columns (Fig. 4b). The space between columns is filled with keratoses sponge remains (Fig. 4c). Abundant ostracods, thin shelled bivalves and subordinate small ammonoids occasionally accumulate between the columns. Dolomite beds are characterized by microcrystalline idiomorphic dolomite rhombs without any bioclast (Fig. 4d, e). The basal Early Triassic Elika Fm. is dominated by finely recrystallized sparry calcite (Fig. 4f). No bioclasts or bioturbation are observed and sparce calcite spheres are rarely visible.

The Abadeh section represents an interior carbonate platform depositional setting, below storm wave base^32^. In contrast the Baghuk section was at the continental margin of a carbonate platform, also below storm wave base^35,36^. According to the same studies, no evidence of significant sea–level change is evident in the studied section.

The conodont biostratigraphic schemes of Yuan et al.^30^ and Korn et al.^37^ are adopted for the Abadeh and Baghuk sections, respectively. The Permian–Triassic boundary is placed at first appearance datum (FAD) of *Hindeodus parvus* following these references.

## Methods

Stratigraphic sections were logged bed by bed, and samples were collected either from fresh carbonate surfaces or from trenches excavated up to 20 cm deep within shale intervals. A total of 137 pulverized samples were analyzed, comprising 83 samples from Abadeh and 54 from the Baghuk section. For each analysis, powders were split from a single homogenized bulk sample. Prior to powder preparation, visible calcite veins, weathered surfaces, large bioclasts, cracks, and stylolites were removed using a rock saw.

### Carbon isotope analysis

Approximately 300 μg of the powdered samples were weighed into 12 mL vials with exetainer caps and loaded into a horizontal rack without caps. About 200 μL of specially prepared anhydrous phosphoric acid was carefully added to the vial neck. Vials were flushed with UHP Helium for 10 min at ~ 70 mL/min, then placed vertically to allow the acid to react with samples. The vials were transferred to a GasBench heated block at 25 °C and left to react for ~ 24 h to release CO_2_ from calcite. The evolved CO_2_ headspace was then analyzed for ^13^C/^12^C ratios. Results are reported in the per mil notation (‰) relative to the international V–PDB scales for δ^13^C. QA/QC was ensured by running the University of Calgary Isotope Science Laboratory internal carbonate standards at the beginning and end of each batch to normalize the data and correct for instrument drift. These standards are periodically calibrated against International Reference Materials to maintain accuracy to the V–PDB scale. Calibration for carbon isotope was based on standards of NBS 18 (5.1 ± 0.1‰), NBS 19 (1.95), IAEA CO-1 (2.5), IAEA CO-8 (5.8‰), IAEA CO-9 (47.1‰), and LSVEC (46.6 ± 0.15‰). Analytical precision and accuracy, based on 1σ of *n* = 10 lab standards were 0.2‰ for δ^13^C_carb_.

### Elemental analysis

Bulk major, trace, and rare element concentrations were determined using ICP–MS. Data quality was verified by international standards and analysis of replicate samples; analytical precision and accuracy are within 10%. Samples were digested using a combination of four high purity acids (HF, HNO_3_, HClO_4_, HCl). Precision and accuracy for elemental concentrations were determined using the reference standards OREAS 101B and OREAS 903, as well as random duplicate analysis of sample splits. The precision and accuracy are within 10% for random splits. The bulk mercury (Hg) concentration was measured using the ICP–MS cold vapor method. Analytical precision and accuracy are within 7%.

To minimize dilution effects from biogenic mineral phases (such as Si and Ca), we normalized trace element concentrations to Al, which is an indicator of the aluminosilicate fraction of sediments and has negligible remobilization during diagenesis. Enrichment factors (EF) were determined using the Tribovillard et al.^38^ formula: EF_element_ X = X/Al_sample_/X/Al_UCC_ in comparison to average upper continental crust (UCC) values^39^.

### Thermal ionization mass spectrometry (TIMS)

The ^87^Sr/^86^Sr isotope ratios were measured for seven samples from the Baghuk section microbiolites and shale intervals using a Thermo–Fisher Scientific Triton Thermal Ionization Mass Spectrometer (TIMS) at the Isotope Science Laboratory (ISL) of the University of Calgary. For ^87^Sr/^86^Sr isotope ratios analysis, 5–10 mg of samples was weighed and 0.5 mL of 1M HNO_3_ was added to the samples. The samples were then centrifuged, and the nitric acid was decanted for ion exchange after eight days of leaching. Prior to the TIMS analysis, the strontium in the samples was isolated using EiChrom Sr resin.

The samples were then loaded onto a single rhenium filament using a Ta_2_O_5_ activator solution. Ten blocks of 15 cycles were measured (for a total of 150 ratios/sample) with matrix rotation of the amplifiers between the blocks. Analytical reproducibility of the ^87^Sr/^86^Sr ratios is + /- 0.000013 (2SD) based on repeated measurement of the standard SRM987 and the duplicate analyses prepared. The measured ^87^Sr/^86^Sr ratio was normalized to the accepted ^88^Sr⁄^86^Sr ratio of 8.375209.

### Programmed pyrolysis

Bulk powder samples (~ 70 mg) were analyzed using HAWK TCO analyzer with the programmed pyrolysis method^40^. The pyrolysis stage (under a N_2_ atmosphere) involved the initial iso–temperature of 300 °C for 3 min to release free hydrocarbons in the samples (S1, mg HC/g rock), followed by increasing temperatures at 25 °C/min up to 650 °C to release hydrocarbons and the oxygen contained in pyrolizable kerogen (S2, mg HC/g rock, and S3, mg CO_2_/g rock, respectively), through thermal cracking. Samples were then automatically transferred to the oxidation oven.

During the oxidation stage, samples were heated from 300 to 850 °C at a rate of 20 °C/min to measure the residual inert organic carbon (S4, mg CO and CO_2_/g rock and residual carbon (RC), wt. %), and a portion of the mineral carbon (MinC, wt.%). Total organic carbon (TOC, wt.%) is quantified as the sum of the total quantity of organic matter released during pyrolysis (Pyrolizable Carbon (PC), wt.%) and the oxidation step (RC wt.%)^40^.

## Results

**Fig. 5:**
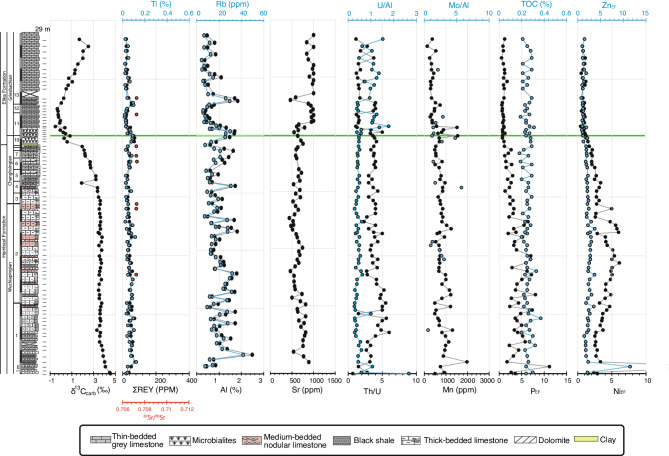
Stratigraphic log, conodont zones, and geochemical data from the Abadeh Section. Green line indicates the biostratigraphic position of the PTB. Strontium isotope data are from^41,43^. Conodont biostratigraphy is from^30^. Numbers on the left of the lithologic log indicate conodont zones: 1- *Clarkina transcaucasica*, 2- *C. orientalis*, 3- *C. wangi*, 4- *C. subcarinate*, 5- *C. changxingensis*, 6- *C. yini,* 7- *C. nodosa*, 8, *C. abadehensis*, 9- *C. hauschkei*, 10- *Hindeodus praeparvus*, 11, *H. parvus*, 12- *Isarcicella staeschei*, 13- *I. isarcica*.

**Fig. 6:**
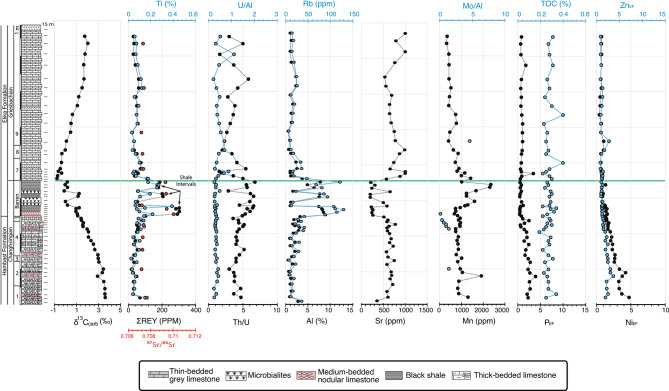
Stratigraphic log, conodont zones, and geochemical data from the Baghuk Section. Green line indicates the biostratigraphic position of the PTB. Conodont biostratigraphy is from^37,44^ Numbers on the left of the lithologic log indicate conodont zones: 1- *C. changxingensis*, 2- *C. Bachmanni*, 3- *C. nodosa*, 4- *C*. *yini,* 5- *C. abadehensis*, 6- *C. hauschkei*, 7- *H. parvus*, 8- *Isarcicella staeschei*, 9- *I. isarcica*.

The early Changhsingian of the Permian Hambast Fm. in the Abadeh section shows consistently high δ^13^C_carb_ values (~ + 3.5‰ VPDB), which decline from the *Clarkina changxingensis* Zone, reaching ~ –0.5‰ at the top of the microbialite interval (Fig. 5), in agreement with published records from this section^41–43^. In the Triassic *Hindeodus parvus* Zone, δ^13^C_carb_ values stabilize around –0.3‰ before a positive shift to + 2‰ in the *Isarcicella isarcica* Zone. Similarly, in the Baghuk section, δ^13^C_carb_ values decreases from + 3.5‰ in the *C. changxingensis* Zone to ~ –0.5‰ in the earliest Triassic *H. parvus* Zone (Fig. 6), followed by a + 2‰ rise in the later Griesbachian.

Throughout the Abadeh section, ΣREE + Y concentration remains low (< 100 ppm), mirroring Ti concentration trends (Fig. 5). In contrast, ΣREE + Y and Ti concentrations increase significantly in Baghuk’s black shales, reaching 400 ppm and 0.4%, respectively, but remain constant in the microbialite beds. Rb and Al concentrations are low in the carbonate rocks of both sections (< 40 ppm and 4%, respectively), however, these values increase dramatically (up to 130 ppm and 8%, respectively) in the back shale beds of the Baghuk Member. Sr concentration values vary between 500 and 1000 ppm in both sections, except for, noticeably low values in the back shale beds of the Baghuk Member.

Permian limestone ⁸⁷Sr/⁸⁶Sr values range from 0.70718 to 0.70766 and Triassic limestones from 0.70716 to 0.70740 in the Baghuk section (Fig. 6). Elevated radiogenic ⁸⁷Sr/⁸⁶Sr (of the Baghuk Member) values (0.70932–0.71006) are always associated with black shales.

At Abadeh, U/Al ratios remain stable (~ 0.5) throughout the Late Permian, through the microbialite layer, and across the extinction horizon, and then rise slightly in the Early Triassic thin–bedded limestones, similar to Baghuk. The Th/U ratio stays around 2 in the Permian microbialite/dolomite unit but declines slightly in Early Triassic limestones. Mo/Al ratios are consistently low (< 6) with no trend in either section.

In the Abadeh section, Mn concentration varies between 500–1000 ppm in the Permian Hambast Fm., with peaks (1500 ppm) in the microbialites, and lows (< 500 ppm) in Early Triassic limestones. In the Baghuk section, Mn concentration trends are similar, but increase to 2500 ppm in the Baghuk Member before stabilizing at ~ 500 ppm in the Early Triassic Elika Fm.

Paleoproductivity proxies (Ni, Zn, P) enrichment factors are high (~ 5) in the Late Permian but decline from the *C. changxingensis* Zone, drop below 1 in the earliest Triassic, and stay low in the Griesbachian in both sections. TOC content remains consistently low (0.2–0.3 wt.%) across all intervals in both sections.

## Discussion

Biostratigraphic and radiometric age determinations show that deep–marine sections with continuous sedimentation are characterized by a gradual and globally synchronous negative trend in δ^13^C_carb_ across the EMPE (e.g.^3,45^). In contrast, shallow–marine sections with erosional unconformity exhibit a sudden drop in the carbon isotope record at the extinction horizon^17,29^. Continuous sedimentation across the PTB in both of our studied sections is supported by: 1) independent biostratigraphic studies, and b) a gradual ∼5‰ decrease in δ^13^C_carb_ values synchronous with other well–dated Iranian^46,47^ and Chinese sections e.g. Shangsi^48^, and also with previous reports from Baghuk^37^. This negative carbon isotope excursion (CIE) starts in the uppermost *C. changxingensis* Zone, consistent with isotopically light CO_2_ released from the Siberian Traps ca. 90 k.y. before the main extinction horizon^49^. The significant riverine and terrestrial input in the black shale beds of the Baghuk section is demonstrated by an abrupt and substantial increase in the concentration of terrestrially derived elements, such as Rb, Al, Ti, and ΣREE + Y (Fig. 6). Elevated ^87^Sr/^86^Sr ratios, reaching extremely high radiogenic values (0.71), exclusively in black shale beds indicate the riverine (terrestrial) and related diagenetic influence on the expected secular trend of the ^87^Sr/^86^Sr curve across the PTB (from 0.7071 to 0.7073), as previously documented in the Abadeh section^43,50–52^ without any siliciclastic lithology. Elevated concentration of Rb (and depletion of Sr), exclusively in the fined–grained black shales, shows the increased flux of aluminosilicate detritus (clays, feldspars, micas) as weathering products^53^. The increase in aluminosilicate detritus is also confirmed by Al concentration in shale beds (Fig. 6). Hence, increased radiogenic Sr due to the in–situ decay of ^87^Rb and recrystallization of carbonate minerals in interaction with pore water (rich in clay–derived Sr) has resulted in the significantly higher ^87^Sr/^86^Sr ratios in the black shales^53^. This condition indicates (terrigenous) sediment bypass through local depressions around lens–shaped microbialite humps (Fig. 3b), which is compatible with development of discontinuous microbial buildup in a platform margin/slope setting^54^. These features support the hypothesis of an enhanced hydrological cycle and intensified weathering rates due to a rapid warming at the extinction horizon^5,6^.

Very low U/Al and Mo/Al ratios as well as high Th/U ratios suggest oxic marine condition during the Late Permian. Paleontological evidence, including diversified ammonoid^55^ and ostracod^56^ assemblages, independently confirm the prevalence of an oxic environment during the Late Permian in the studied area. The persistence of the same geochemical trends across the EPME, and throughout the black shale and microbialite intervals in both sections suggest the prevalence of oxic condition during the EPME in our studied sections (Figs. 4 and 5).

Mn concentration (Mn/Sr) is commonly used as an indicator for diagenetic alteration and generally expected to be elevated in diagenetically altered samples. However, Mn concentration can be strongly influence by environmental conditions and can reflect paleoredox condition rather than diagenetic alteration. There are several studies that focus on the primary vs. diagenetic origin of high Mn concentration and indicate the importance of redox conditions rather diagenetic alteration (for Early Triassic example see^57^). Mn(II) is soluble but readily oxidizes to form Mn(IV) particles if any free O_2_ is available in the water column. These particles sink down in the water column and the Mn is remobilized in reducing water or sediments, which induces dynamic Mn cycling at redox interfaces^38,58^. Mn enrichment then occurs along the redox interface between anoxic Mn^2+^ enriched waters and oxic surface waters and has been shown as a robust proxy for reconstructing the redoxcline position^59^. The enrichment of Mn in both sections, in the Baghuk Member (immediately below PTB), is contrary to previously reported Mn depletions in deep slope (Chibi, China), distal ramp (Chanakhchi, Armenia) settings of the Tethys^19^, as well as in the deep Panthalassa Ocean^20^. Grasby et al.^20^ showed that these Mn depletions reflect deep ocean anoxia mobilizing Mn^2+^ into seawater. The Mn enriched horizons reported here suggest then an effective shuttle to the redox interface which must have been close to the water depth of the sections studied. The position of the chemocline likely fluctuated, e.g. due to variable water transparency–related light conditions^60^, in relation to the collapse in productivity^61^, and global transgression^59^ at that time, allowing episodic influx of Mn^2+^ into the dominantly oxic environment, similar to the ore deposit model of Frakes and Bolton^62^. A temporal episode of Mn enrichment only within the extinction interval, together with a uniform increase in Mn concentrations in both the black shales and the microbialite beds, with and without evidence of terrestrial input, respectively, rules out a terrestrial origin for the Mn. According to this scenario, the significantly elevated Mn concentration in the Central Iranian (Tethyan) shelves likely originate from anoxic deep ocean water with depleted Mn concentration^20^. Upward diffusion of Mn^2+^ from the deep ocean into the oxic shallow settings is the more likely scenario compared to diagenetic alteration^57^. Episodic deoxygenation across the EPME in the eastern Tethys (S. China) due to enhanced ventilation and a decrease in primary productivity is documented by Yang et al.^63^. The variable influx of Mn hints at episodic fluctuations of the chemocline repeatedly drowning this habitat in the anoxic water column. Herwartz and Viehmann^60^ suggest that chemocline depth in a stratified ocean may range between 200 and 3 m depth depending on nutrient supply and light conditions. Daily 40 m vertical movement of the redox boundary is observed in the Black Sea^64^. Whether or not fluctuations on daily and seasonal timescales could potentially provide an efficient kill mechanism via suffocation thus remains unclear. According to Frank et al.^23,65^ the presence of ostracods, bivalves, gastropods, and trace fossils in shallow–marine setting of the western Tethys (Dolomites) across the EPME indicate that the episodic deoxygenation was not sufficient to cause local extinction, suggesting that anoxia was not the only kill mechanism during the EPME in shallow marine environments. Due to their transient nature, such events would not necessarily be recorded in the geological record and even if they are, the water column close to the sea–air interface and in the vicinity of O_2_ producing microbial mats would remain oxygenated.

A slight but noticeable decrease in Th/U and Mn, associated with an increase in U/Al ratios above the microbialite and black shale intervals (*H. parvus* and *I. isarcica* Zones) indicates less oxygenated (dysoxic) condition in the earliest Triassic. This is in good agreement with the rising sea–level and a general ascent of the chemocline in the studied area during the earliest Triassic^32^. Thin-section observations also show that fossil abundance falls dramatically at the contact between the microbialite and the overlying thin–bedded limestone, when the O_2_ producing microbial mats vanished. This shows that the main decline in abundance of metazoans is not synchronous with the onset of microbialite deposition in the studied area. This may indicate that the driving mechanism fostering the takeover of microbialites, which is either biological (absence of grazers) or environmental/climatic (supersaturation/warming), were different than the drivers of metazoan abundance decline. Hence, different stressors at the EPME (not only anoxia) most likely caused different environmental/biological impacts and different phases of the extinction, indicating the complexity of the EPME.

Using TOC content alone as a productivity proxy can be ambiguous due to preservation bias, but micronutrients such as Ni, and P content can also serve as paleo–productivity proxies^38,66–68^. The decline of these proxies in both sections suggests diminishing primary productivity synchronous with the δ^13^C_carb_ negative excursion starting in the *C. changxingensis* Zone, well before the main extinction horizon. The decline in primary productivity during the Late Permian is also documented in both the eastern and central Tethys^68^, implying disruption of the food web and, hence, affecting higher trophic levels when culminating at the EPME. This observation agrees with nutrient trapped in anoxic deeper waters, leading to oligotrophic surface waters in the equatorial Tethys^13^. The synchronicity of collapse in primary productivity and negative δ^13^C_carb_ excursion in the studied area, suggest that dissolved inorganic carbon increased in the surface ocean^12^. Low paleoproductivity can indicate that O_2_ demand for remineralization of OM was low at the PTB in the equatorial Tethys and that the O_2_ deficiency and anoxia had been less severe compared e.g. to the Panthalassa Ocean, locally providing a refugia for O_2_–metabolizing marine species to survive in low oxygen condition. By similar reasoning, Yang et al.^63^ related the renewal of oxic conditions to a decline in primary productivity in S. China.

Geochemical evidence in the two studied sections indicate that mostly oxic conditions were repeatedly disrupted by expansion of anoxic conditions. Two principal sources of dissolved O_2_ that may preserve habitable oxic microenvironments during severe anoxic events include 1) molecular oxygen derived from the atmosphere, and 2) O_2_ produced by photosynthesizers. In the modern ocean, the relative contribution of these two sources to dissolved oxygen can be quantified due to their distinct triple oxygen isotope (Δ^17^O) fingerprints^69^. Efficient gas exchange is facilitated by windy conditions and episodic algal blooms resulting in variable proportions generally dominated by atmospheric O_2_^70^. Notably, a recent study by Buchwald et al.^71^ documented phytoplankton blooms at the PTB in mid- to high-latitude settings (Svalbard and Italy), which could also be considered an oxygen source. An interesting example of the significance of cyanobacteria in providing oxygenated conditions within an entirely anoxic environment, is the formation of O_2_ rich oases by accumulation of photosynthetic O_2_ before the Great Oxygenation Event^72^. In addition, paleontological and geochemical studies have emphasized the role of active production of O_2_ (and food) by cyanobacterial mats during the EPME^73^, as well as the oxygenated depositional environment of PTB microbialites, as documented by other studies^23,74,75^. The association of bioturbation structures, bivalves, and echinoids with microbial mats on the deep slope in the Shangsi section^76^ implies that microbial mats may have acted as refugia during the extinction event. However, gas exchange with the atmosphere will always represent a significant if not dominant source of dissolved O_2_, especially in wave dominated shallow–marine environments, which could have inhabited diverse benthic fossil assemblages^77^. Although several benthic communities have been reported from the EPME microbialite interval^29,75,76,78,79^, the concentration of fossils in cavities and the intervening spaces between microbialite structures suggests that the microbialite morphology may have played a greater role in the physical preservation of transported fossils rather than in providing a truly oxygenated environment caused by cyanobacteria activity^74^. Moreover, modern microbial mats are known to oxygenate only a few millimeters of their immediate surroundings^80,81^, implying that any oxic conditions would have been restricted to a thin envelope, if they were the sole source. However, it remains unresolved whether photosynthesis or dissolved atmospheric O₂ was the primary oxygen source. A quantitative understanding of the relative proportion of photosynthetic O_2_ vs. atmospheric O_2_ can be attained by modelling gas exchange and photosynthesis and/or by analyzing Δ^17^O of dissolved O_2_ in surface waters of modern anoxic lakes, but this is beyond the scope of this study.

## Conclusion

The shallow–marine area of the equatorial central Tethys Ocean was well–oxygenated during the Late Permian. This oxic condition persisted in the microbialite and black shale intervals. The shift toward anoxic conditions in the earliest Triassic oxic habitats was driven by sea-level rise and potentially the absence of cyanobacteria. It remains unsolved if the main source of dissolved O_2_ was provided by the atmosphere or by cyanobacteria. In addition to photosynthetic activities, wind and wave action facilitates atmospheric O_2_ dissolution in shallow water. Notable changes in redox proxies toward anoxic condition with the rise in sea–level indicate that the oxic–anoxic boundary is linked to water depth, implying only transient anoxia. The low local paleoproductivity and OM burial indicate that remineralization of OM did not largely affect the oxygenation state. This study shows that perturbation in the carbon cycle, collapse in paleoproductivity, and the EPME occurred in a predominantly oxygenated environment in the central Tethys. Rapid fluctuations of the chemocline as evidenced by particulate Mn enrichment suggests the repeated invasion of Mn–reducing water masses from deeper waters, exerting pressure on the benthic community, leading to a dramatic loss of biodiversity at the EPME.

## Supplementary Information

Supplementary Information 1.

Supplementary Information 2.

Supplementary Information 3.

Supplementary Information 4.

## Data Availability

Data is provided within the manuscript or supplementary information files.
