# Supplementary figures and images for: Habitability at the edge of the redox boundary during the Permian–Triassic mass extinction
Source: Sci Rep. 2026 Apr 15;16:12469. doi: 10.1038/s41598-026-47893-w (PMC13087033; doi:10.1038/s41598-026-47893-w)

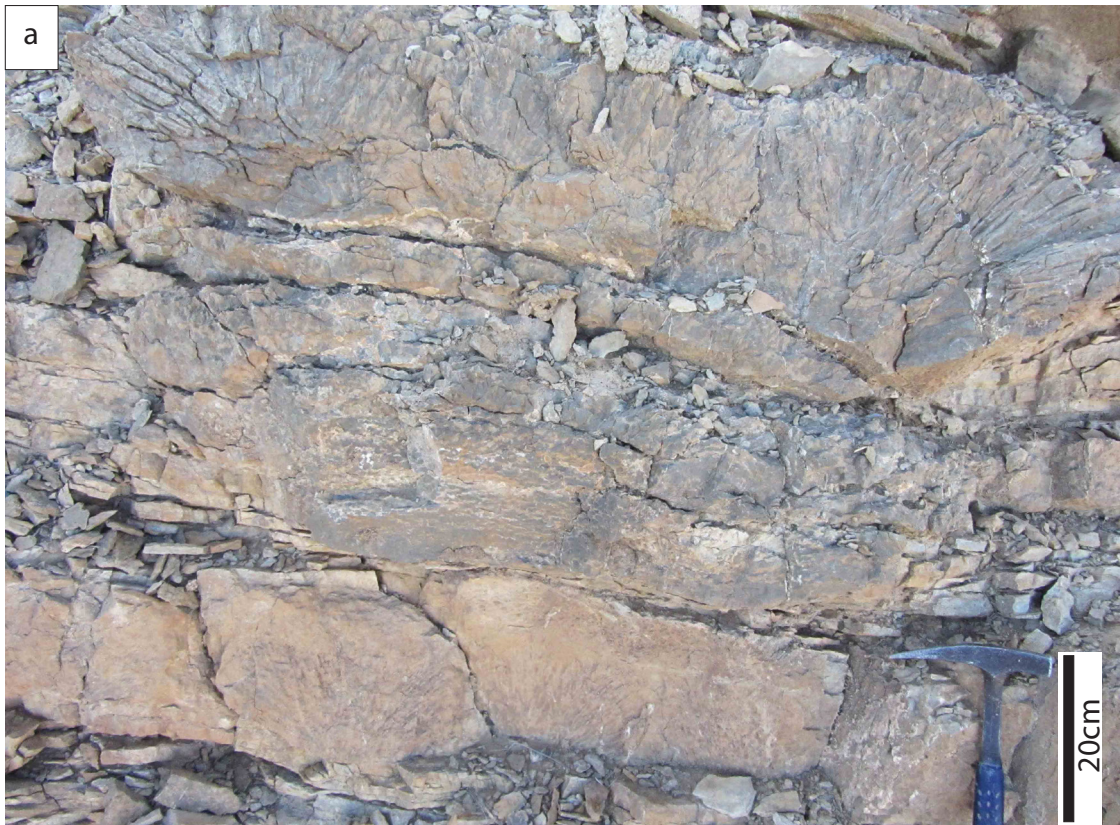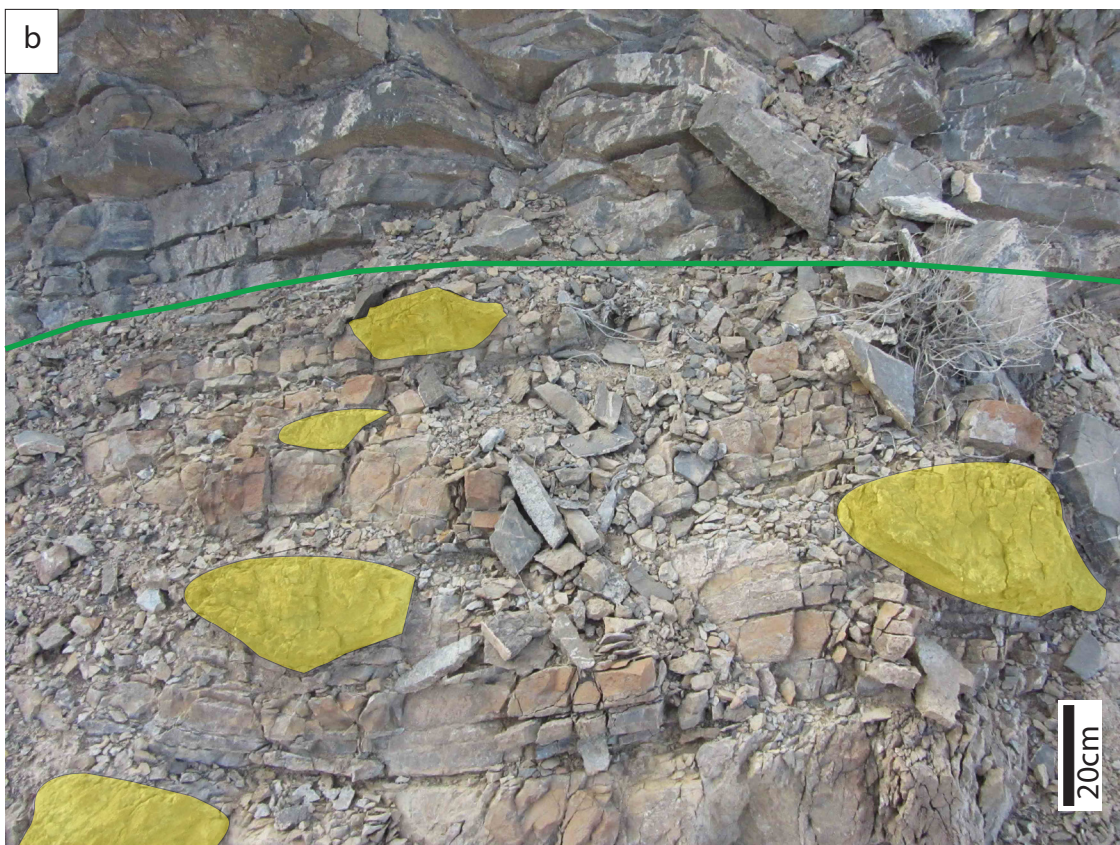

Supplemental Figure S1

Supplement: Supplementary file 1 — Supplementary Information 1. [file 41598_2026_47893_MOESM1_ESM.pdf]
