# Supplementary material for: Habitability at the edge of the redox boundary during the Permian–Triassic mass extinction
Source: Sci Rep. 2026 Apr 15;16:12469. doi: 10.1038/s41598-026-47893-w (PMC13087033; doi:10.1038/s41598-026-47893-w)
Supplement: Supplementary file 4 — Supplementary Information 4. [file 41598_2026_47893_MOESM4_ESM.docx]

**Supplementary materials caption:**

**Table S1.** Geochemical data for samples from the Abadeh section.

**Table S2.** Geochemical data for samples from the Baghuk Section.

**Figure S1. a,** Microbialites in the uppermost part of the Hambast Fm. in the Abadeh section. **b,** intercalating microbial build ups (transparent yellow areas) with dolostone bed in the uppermost part of the Hambast Formation in the Abadeh section. The Green line marks the base of the Elika Fm.
